# Mandibular Canine Transmigration: A Case Report

**DOI:** 10.7759/cureus.90781

**Published:** 2025-08-22

**Authors:** Rajashree Bhattacharjee, Kuldeep Phukon, Arpita Kashyap

**Affiliations:** 1 Orthodontics and Dentofacial Orthopedics, Government Dental College, Silchar, Guwahati, IND; 2 Orthodontics and Dentofacial Orthopedics, Regional Dental College, Guwahati, Guwahati, IND

**Keywords:** biomechanics, cone beam computed tomography, dental anomaly, impaction, intra-osseous, transmigration, transplantation

## Abstract

This article aims to illustrate a case of mandibular canine transmigration crossing the midline and erupting labially to the lower incisors and discuss its general prevalence, etiology, and orthodontic management. Cases of canine transmigration are rare and mostly occur due to retained deciduous predecessors or dental lamina aberrations. This anomaly is appropriately diagnosed radiographically. As canines are significant for masticatory function, arch stabilization, and esthetics, it is imperative to preserve them by orthodontic correction or surgical transplantation, unless the prognosis is appalling. A 13-year-old girl presented with extra erupting teeth in the front lower teeth region. A transmigrated 43 across the mandibular midline erupted labially to 31, confirmed by orthopantomogram and cone beam computed tomography. Thirty-three erupted mesially and labially to 32. Treatment involved the extraction of retained 83 and transplantation of 43, along with the application of traction force to bring 33 into its anatomical position.

## Introduction

Transmigrated teeth are those that show intra-osseous movement within the bone across the midline before they erupt ectopically in positions different from their anatomical ones. Though not a very common phenomenon, it is mostly encountered in the mandible rather than the maxilla, more commonly in female patients. More often than not, this anomaly affects the permanent canine tooth. Diagnosis of such a case is generally made during routine or diagnostic radiographic evaluations. The etiology of such cases is generally not specific, although some suggest genetic factors, persistent deciduous teeth, endocrine disorders, dental lamina position aberrations, cystic lesions, etc., as some probable etiologies.

Canines are responsible for a good functional occlusion and a fully esthetic smile. So when a case of transmigrated canines is stumbled upon, ideally, the orthodontic treatment plan should aim at restoring the normal position of such teeth. However, it may not always be prognostically favorable to do so, especially when the position of the canine is extremely critical or submerged in the bone or completely horizontal, etc. Under such circumstances, canines are kept under observation unless they become symptomatic, in which situation they are surgically removed.

A classification of transmigrated mandibular canines was given by Mupparapu [1] in 2002, depending upon the ultimate placement of canines within the jaw and their migration path.

## Case presentation

**Figure 1:**
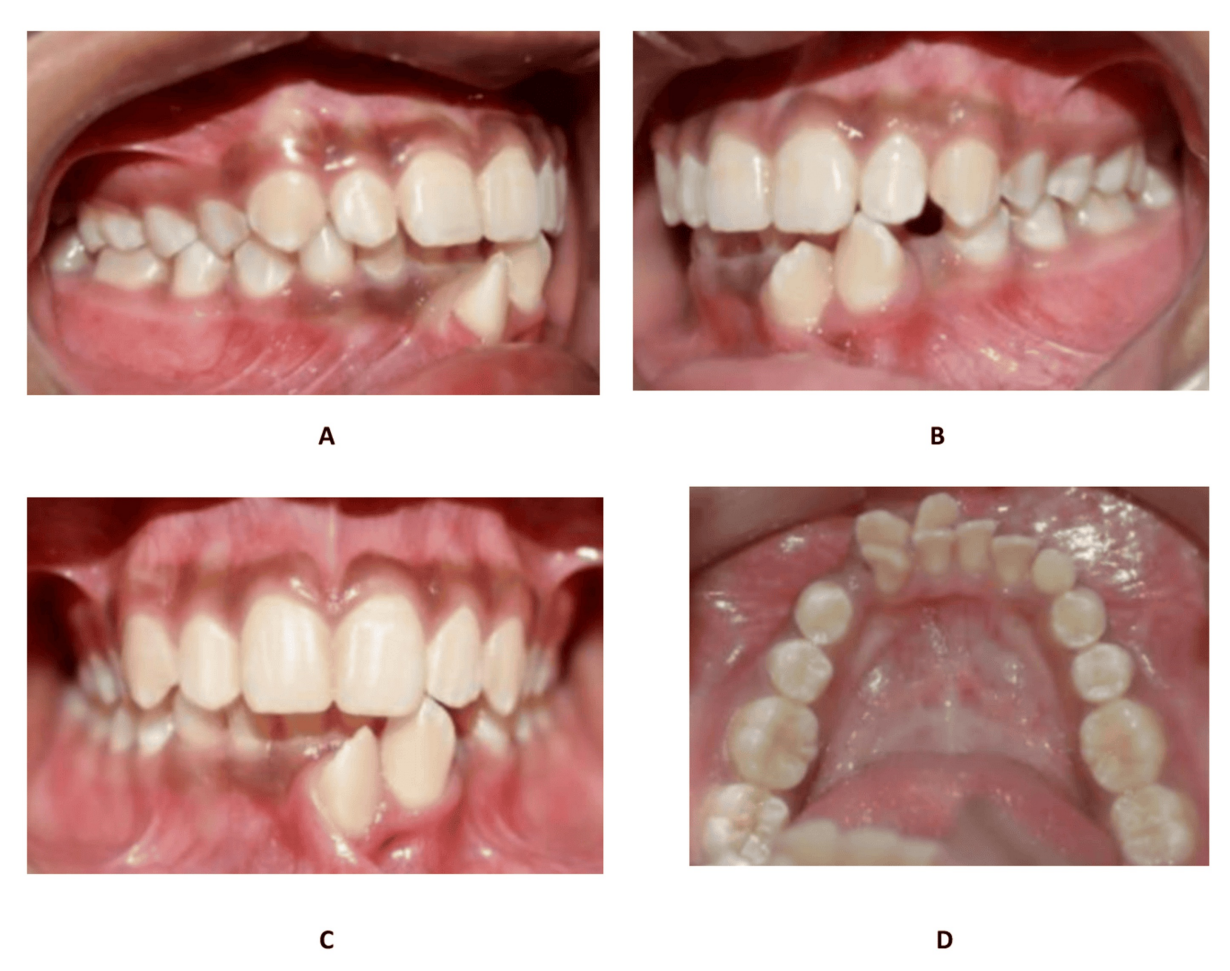
Pre-treatment intraoral photographs (A) Maxillo-mandibular occlusal relation - right side. (B) Maxillo-mandibular occlusal relation - left side. (C) Maxillo-mandibular occlusal relation -frontal view. (D) Mandibular occlusal view.

**Figure 2:**
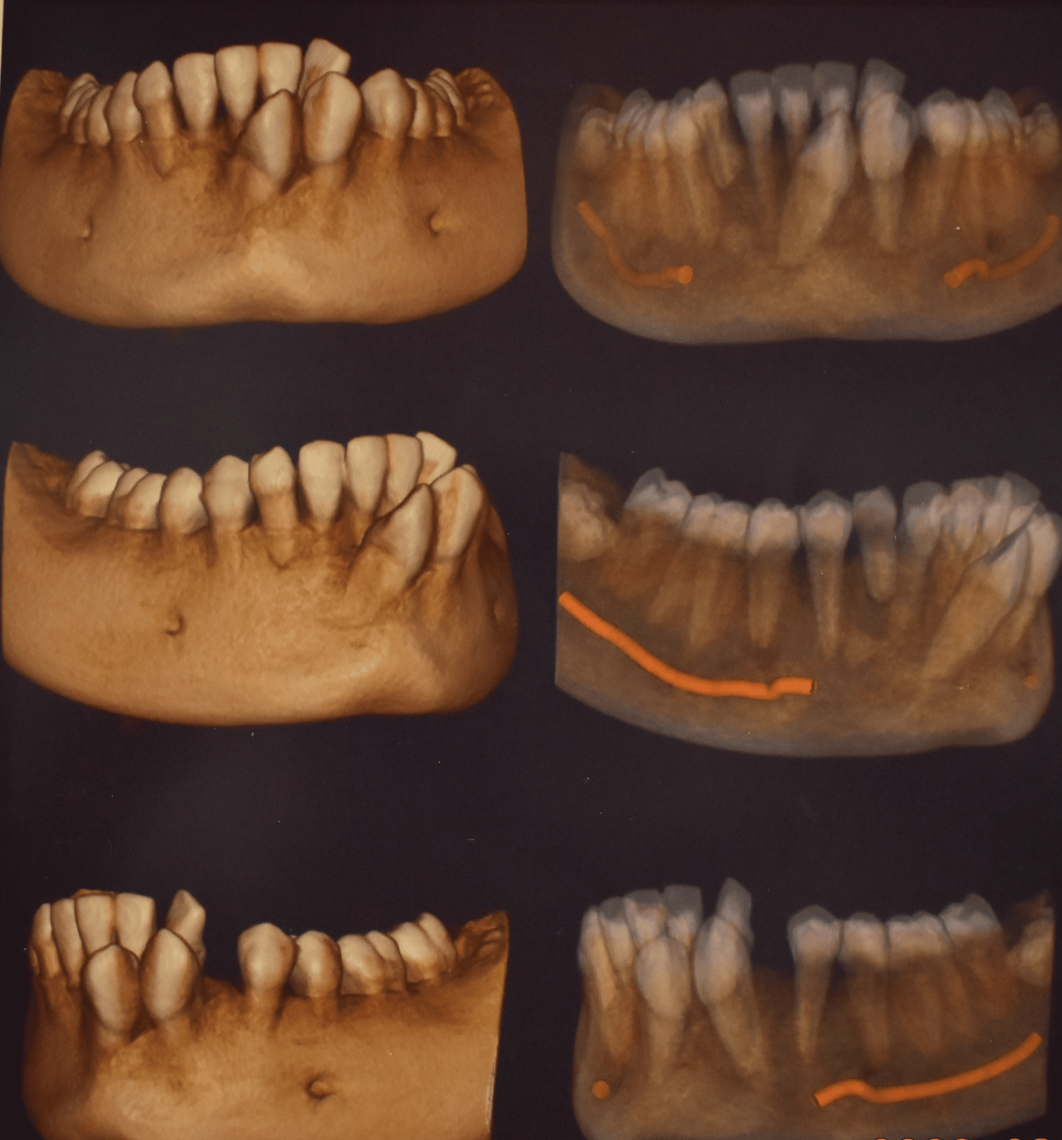
Pre-treatment CBCT imaging of the mandible CBCT: cone beam computed tomography

**Figure 3:**
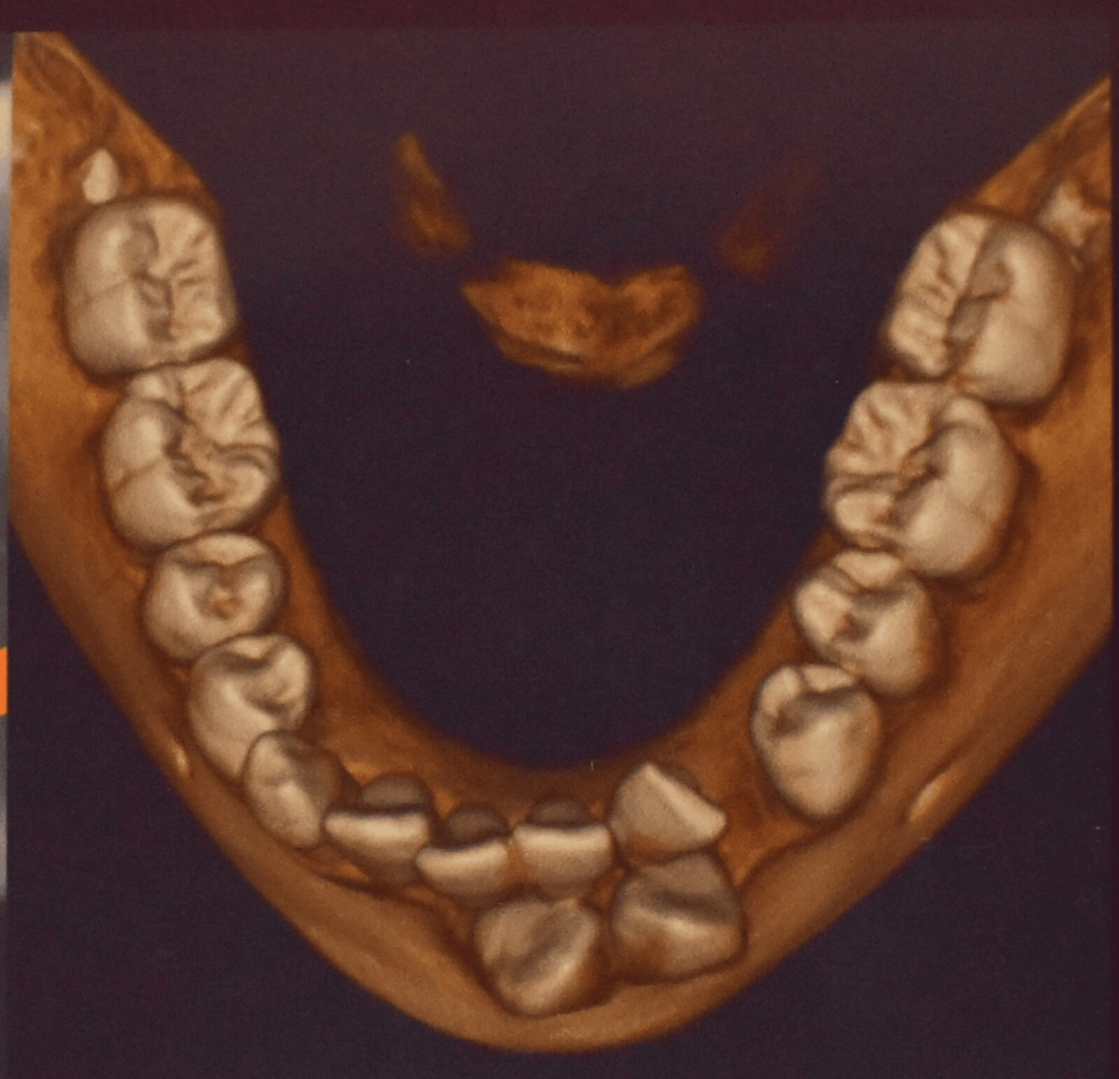
Occlusal view CBCT imaging of the mandible CBCT: cone beam computed tomography

The article presents the case of a 13-year-old female Assamese patient (with a Mongoloid origin) reporting with a complaint of extra erupting teeth in the front lower teeth region. The patient's medical history was not significant. Extraoral examination revealed no malformations or any facial asymmetry. The patient had a convex facial profile with incompetent lips. An intraoral examination showed the presence of a retained 83 and spacing present in the 33 region (Figure 1). What appeared to be right and left mandibular permanent canines were present in the lower midline area near the incisors. Mild lower anterior teeth crowding was present. The molar relation was Angle’s class I bilaterally. The panoramic radiograph and cone beam computed tomography (CBCT) images, along with the clinical presentation, confirmed the presence of a transmigrated 43, which was positioned more mesial and labial to 42, crossing the mandibular midline, and placed labially opposite to 31 (Figures 2-3). Additionally, it was seen that the left mandibular canine, 33, erupted mesiolabially to 32. With a little mesial rotation, the cusp tip of 33 was situated 4.4 mm below the incisal surface of 32, creating a mirror image of 43. These presentations of both the mandibular permanent canines strongly suggested type 3 transmigration of the canine 43 according to Mupparapu [1]. The patient was a horizontal grower with a class I skeletal relation of the upper and lower jaws.

After the preliminary investigations, the treatment plan devised for the patient was to extract 83 and transplant the transmigrated 43 at its anatomical position, i.e., auto-transplantation of 43. Meanwhile, 33 was to be brought back to its anatomical position by traction force. The appliance used was a 0.022-inch slot pre-adjusted edgewise MBT appliance. Once 83 was extracted, the oral surgeon performed the extraction and transplantation of 43 into the socket of 83 at the same appointment. Leveling and alignment were started on a 0.012-inch nickel-titanium (NiTi) archwire. Canine laceback was applied on 33 to exert a traction force, pulling it into the anatomical position. The upper arch was similarly leveled and aligned with NiTi archwires. Traction of 33 into the anatomical position was finished, and the arches were stabilized with 0.019 x 0.025-inch stainless steel archwire. Intentional root canal treatment of 43 was done to avoid any future root resorption or mobility. Finishing and detailing of the arches was done using 0.016-inch beta titanium archwire.

**Figure 4:**
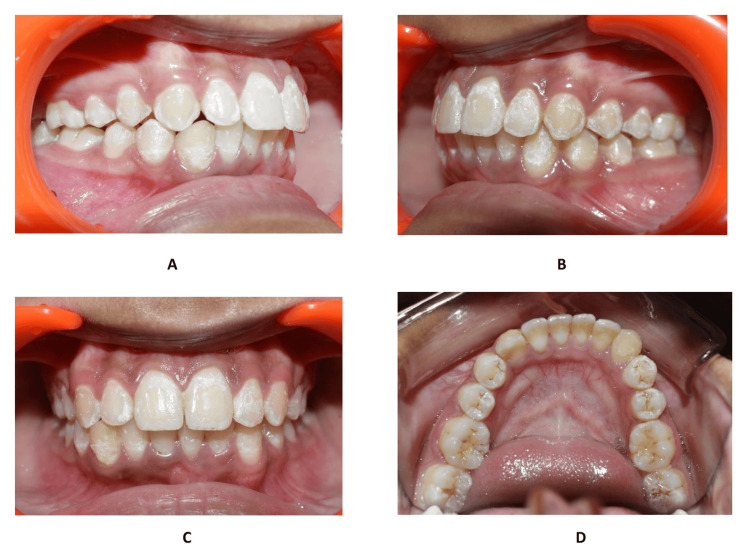
Post-treatment intraoral photographs (A) Maxillo-mandibular occlusal relation post-treatment - right side. (B) Maxillo-mandibular occlusal relation post-treatment - left side. (C) Maxillo-mandibular occlusal post-treatment - frontal view. (D) Mandibular occlusal post-treatment view.

Finally, debonding of the appliance was followed by a fixed retainer in both upper and lower arches, as well as a removable Hawley’s retainer (Figure 4). After a surgical consultation, the transpositioned canine was left for further radiological observation.

## Discussion

Dental transmigration is an infrequent anomaly to come across; the number of reported cases is also low. The incidence of transmigration is typically seen in an impacted mandibular canine rather than in an impacted maxillary canine. A study to investigate the prevalence of canine transmigration in a South Indian population [2] showed the prevalence to be 0.46%, with females reporting more cases than males. Another study in a North Indian population by Sharma and Nagpal [3] showed the overall prevalence of transmigrated canines (15 mandibular and five maxillary) to be 0.66%.

Ando et al. [4] were the first to use the term "transmigration." Transmigration, as defined by Tarsitano et al., is the migration or relocation of an impacted canine, usually mandibular, across the midline of the dentition [5].

Some of the probable causative factors pointed out by a few [4,6,7] are crowded dentition, presence of supernumerary teeth, prolonged retention or premature loss of primary teeth, and even increased crown length of the canines. Peck [8] cited the role of genetics in the etiology of ectopic mandibular canines. The path of eruption and presence of pathologies along the path of eruption, such as cysts, tumors, or odontomas, could also contribute to the occurrence of transmigration [4,6]. Al-Waheidi [9] had pointed out the association of a cystic lesion with transmigrated canines. Hypodontia and excessive spacing in the dental arch have also been linked to the incidence of transmigration of teeth.

Treatment options proposed for a case of canine transmigration vary from a conservative orthodontic alignment to auto-transplantation to surgical removal in extreme cases. Surgical removal is generally the most favored option, especially when the ectopia is critical or the arch has pre-existing crowding. Auto-transplantation is a rare treatment plan option, having an uncertain prognosis in the long run. That was the reason an intentional root canal treatment was done on the tooth to compensate for any future resorption or mobility. Orthodontic alignment, if possible, could undoubtedly be the best option available, provided the path of the transmigrated tooth is not complex. If associated with any pathology, correction of the pathological factor could also improve the prognosis to a great extent, as was reported by Taguchi et al. [10]. The wise old saying of observation for an unerupted transmigrated tooth, unless the patient becomes symptomatic, is also advocated by many [9]. However, intervention should be initiated in any form, such as surgical extraction, in the event of deteriorating conditions of the impacted tooth or cystic transformation.

## Conclusions

Canine transmigration is an unusual and rare event among dental developmental disorders. Its diagnosis is mostly accidental, but the probability of that is now much better due to CBCT in addition to the routine radiographic examinations. Treatment of such a case is usually complicated and rarely leads to an ideal result. Nevertheless, any kind of intervention is initiated based on a precise diagnosis, severity of angulation, and location of the transmigrated tooth as measured from its ideal position in the dental arch. Studies on larger scales and substantial case reports of such anomalies could help improve the diagnosis, determine etiologic parameters, and manage transmigrated teeth.
